# Optimization of Steam-Curing Regime for Recycled Aggregate Concrete Incorporating High Early Strength Cement—A Parametric Study

**DOI:** 10.3390/ma11122487

**Published:** 2018-12-07

**Authors:** Asad Hanif, Yongjae Kim, Muhammad Usman, Cheolwoo Park

**Affiliations:** 1 Department of Civil Engineering, Mirpur University of Science and Technology (MUST), Allama Iqbal Road, Mirpur AJ&K 10250, Pakistan; ahanif@connect.ust.hk; 2 Department of Civil Engineering, Kangwon National University, 346, Jungang-ro, Samcheok-si, Gangwon-do 25913, Korea; 3 School of Civil and Environmental Engineering, National University of Science and Technology (NUST) Sector H-12, Islamabad 44000, Pakistan; m.usman@kaist.ac.kr

**Keywords:** recycled aggregate, steam curing, waste recycling, concrete, mechanical properties, high early strength

## Abstract

This paper investigates the properties of steam cured recycled aggregate concrete (RAC), in an attempt to determine the optimum conditions of the steam-curing cycle for RAC, and incorporating high early strength cement (HESC). Varying conditions of steam curing were employed. The steam-curing cycle was set based on the peak temperature, and the duration for which the peak temperature was maintained. Three peak temperatures were used for steam curing, 50 °C, 60 °C, and 70 °C, maintained for up to two hours. The compressive strength results indicated that the steam-curing cycle employing the peak temperature of 50 °C maintained for one hour with a total duration of four hours was the optimum for strength development, both at the early and late stages of hydration. Determining the optimum steam-curing temperature and duration will help reduce the associated curing cost, thus further economizing the production cost of recycled aggregate concrete.

## 1. Introduction

The use of recycled aggregates (RA) in concrete has gained much attention over the previous several decades, due to continuously declining natural resources leading to increasing interest in sustainable development [1,2,3,4]. This has led researchers to explore various properties, including mechanical properties [5,6,7], durability attributes [8,9,10,11], and dimensional stability [12,13,14], which have demonstrated the efficient utilization of RA for buildings and construction applications. However, it has been shown that using RA in lieu of natural aggregates (NA) reduces the mechanical strength of recycled aggregate concrete (RAC) [15]. However, this reduction is meager when the replacement level is less than 20%. This is of particular concern when the RAC application is in structural and precast construction.

In order to address the aforementioned issue, various methods have been employed to date to improve the strength of the resulting concretes. These include incorporating nanomaterials within the concrete under consideration [16,17,18], using warm water for concrete mixing [19], and/or employing hot temperature steam curing [20,21,22]. However, the health hazards associated with nanomaterial incorporation cause reluctance to its use in concretes. Further, the resulting, minimal strength enhancement with the use of warm/hot water for concrete mixing discourages its application in the precast construction industry, where the objective is faster construction and the speedy erection of the structural elements. This leaves only the steam-curing method as a viable option for the precast concrete industry.

Due to the faster hydration rate and higher strength gain, steam curing has gained wide acceptability. Various research findings have been reported in the past, demonstrating its usefulness in precast concrete manufacturing. Turkel and Alabas [20] conducted an extensive study on various curing temperatures and durations, and showed that 85 °C is the optimum temperature to obtain a high early strength of concrete. Later, Park et al. [21] demonstrated the improved early-age strength of high performance concrete through steam curing. Similarly, in other research, the usefulness of steam curing in enhancing early-age compressive strength has been explored [23,24]. It has been clearly demonstrated that excessive exposure to high-temperature steam curing results in negative effects on mechanical properties [20,25].

Among various research findings published in the past, the work of Ramezanianpour et al. [26,27] is of much significance. They evaluated the effects of different steam-curing regimes on the strength and durability of self-compacting concrete.

Compressive strength measurements indicated that in a constant total time, increase in pre-curing period leads to lower immediate compressive strength. On the other hand, increase in treatment temperature and total cycle time (which means higher energy and time consumption) led to higher immediate compressive strength [26].

The durability results established that the peak temperature of 70 °C imposes a negative effect on durability properties. However, it was seen that they employed very long curing cycles (from 16 h to 20 h), which makes the resulting concrete energy intensive, rather than energy efficient. In the same way, Ba et al. [28] investigated the effects of steam curing on the strength and porous structure of concrete with a low water/binder ratio. They demonstrated experimentally that high temperature steam curing benefits the strength attributes of such concretes, while the exposure duration further augments such characteristics, for up to 14 h total duration of the curing cycle.

Although the useful effects of steam curing on the mechanical properties have been explored in the past, there has been little work conducted evaluating the optimum parameters for steam curing. Moreover, there are no research findings that report the optimum steam-curing parameters for recycled aggregate concrete (RAC). This parametric study fills these research gaps by investigating the effects of various temperatures and exposure durations on the mechanical strength of steam-cured recycled aggregate concrete. The determination of the optimum temperature and exposure duration will lead to reduced costs and embodied energy, thus contributing to sustainable development.

### Research Significance

A number of published studies have comprehensively evaluated the suitability of steam curing in normal concrete, but these were only focused on concrete produced with ordinary Portland cement (OPC) as the binder, and merely followed the steam-curing cycle as recommended by [29]. With the advent of modern construction practices and state-of-the-art techniques, precast construction specialized binders (like high early strength cement, HESC), have attained extreme importance. Evaluating the resulting concrete properties in these scenarios is imperative. Although RAC properties under steam curing have been evaluated previously, the studies on the determination of the optimum steam-curing conditions (like peak temperature, cycle length, and the holding duration of the maximum temperature) are sparse. Further, the properties of RAC made with HESC have not yet been determined. This demands an in-depth evaluation of the RAC properties containing HESC, and the determination of the optimum parameters for the steam-curing cycle.

This experimental study fills the current research gap by exploring various properties of steam-cured, high early-strength RAC. The steam-curing conditions were also varied, so that the steam-curing conditions for optimum concrete properties could be ascertained. The novelty of this study lies in the determination of the optimum steam-curing parameters for recycled aggregate concrete, incorporating high early strength cement.

## 2. Materials and Experimental Methods

### 2.1. Raw Materials

The primary binders used in this study included ordinary Portland cement (OPC) and moderately high early strength cement (HESC).

**Figure 1:**
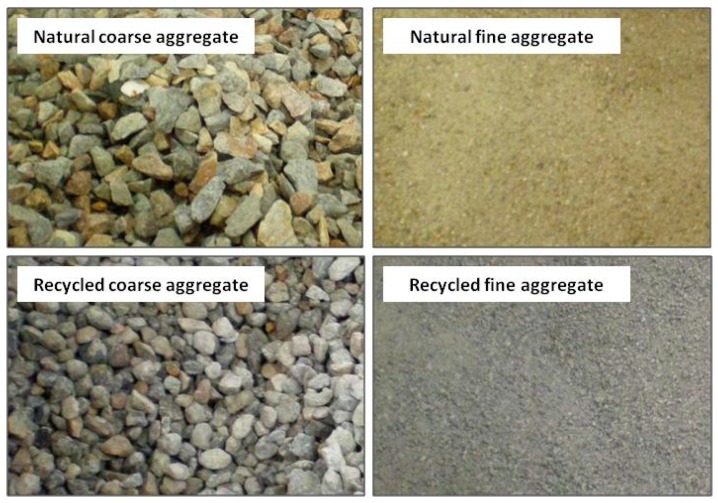
Aggregates used in the experimental work.

The corresponding specific gravity of the OPC and HESC was 3.14 and 3.17, with a Blain surface area of 3340 cm^2^/g and 4490 cm^2^/g, and a loss on ignition of 2.26% and 1.05%, respectively. Their other properties conformed to the Korean Standards KSL5201-1989. Coarse and fine aggregates (both natural and recycled; Figure 1 used in the experiment were graded according to the Korean Standards [30,31,32].

The maximum size of the coarse aggregates (both NA and RA) was 25 mm, whereas well-graded fine aggregates under 5 mm particle size were used in the experimental study. The aggregates recycled by the Insun recycling plant (Ansan, Korea) were used after treatment/bonded mortar removal. This was achieved using several methods, involving “mechanical processes (involving grinding/churning/sieving), thermal processes (involving microwave or conventional heating) and chemical methods (pre-soaking or cyclic soaking of the recycled aggregates in chemical solutions). Up to 95% of the bonded mortar could be successfully removed. Admixtures (high rate water reducing admixture and air-entraining admixtures) were utilized for maintaining the uniformity, consistency, and cohesiveness of the fresh concrete mix while stabilizing the air bubbles in the mix and improving its workability. Ordinary potable tap water (free from impurities and chemicals) was used for mixing the ingredients” [22].

**Figure 2:**
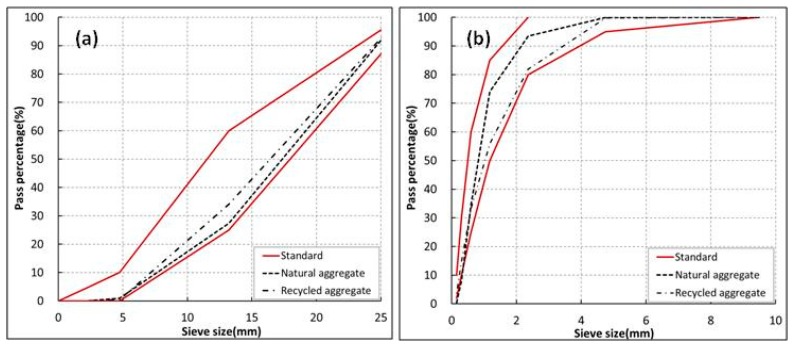
Particle size distribution curve for (**a**) coarse aggregates, and (**b**) fine aggregates.

The basic properties of the raw materials are reported in Table 1 and Figure 2. These properties helped to determine a suitable mix-proportion (Table 2) for design strength and workability requirements. These tests were conducted under the guidelines of Korean Standards (KS) [30,31,32,33]. The testing included gradation, fineness modulus, water absorption, and specific gravity.

### 2.2. Mixture Proportioning and Specimen Casting 

The mix design procedure and concrete mixing, casting, and curing were done according to American Concrete Institute (ACI) standards [34,35,36], and explained by the authors in previously published findings [15,22,25,37] and are hence not repeated here. The mix proportions are given in Table 2. The design strength was 30 MPa while the minimum target slump was set to 150 mm (aggregates were assumed in saturated surface dry (SSD) conditions in the mix formulation procedure, and water adjustment was made later based on the stock condition). As indicated in Table 3, three main series of concrete mixes were formulated. First, containing HESC with all natural aggregates (both coarse and fine aggregates); second, containing HESC with 40% NC (natural coarse aggregate) and 60% NF (natural fine aggregate); and finally, containing OPC with 40% NC and 60% NF. The remaining 60% and 40% aggregates used were recycled coarse and fine aggregates, respectively. The aforementioned percentages of aggregates were based on previous studies [22,25,38,39,40,41], which led to the determination of the optimum performance-based (strength and durability) blend of aggregates (natural and recycled). The series with all natural aggregates was used as a control for comparison purposes.

The concrete was mixed in a rotating drum type mixer. First, all of the required coarse aggregate was put in the mixer along with 50% of the cement. These were mixed for one minute while progressively adding 50% of the required water. After one minute, the fine aggregate, remaining water, and cement were added while the mixer continued to rotate for three more minutes. Admixtures were added simultaneously with the water. The whole mixing procedure took around four minutes. Subsequently, the specimens were cast in molds of various sizes. Cylindrical specimens with a diameter of 100 mm and a length of 200 mm were cast, for compressive strength testing [42]. 

### 2.3. Steam-Curing Conditions Employed in the Study

**Figure 3:**
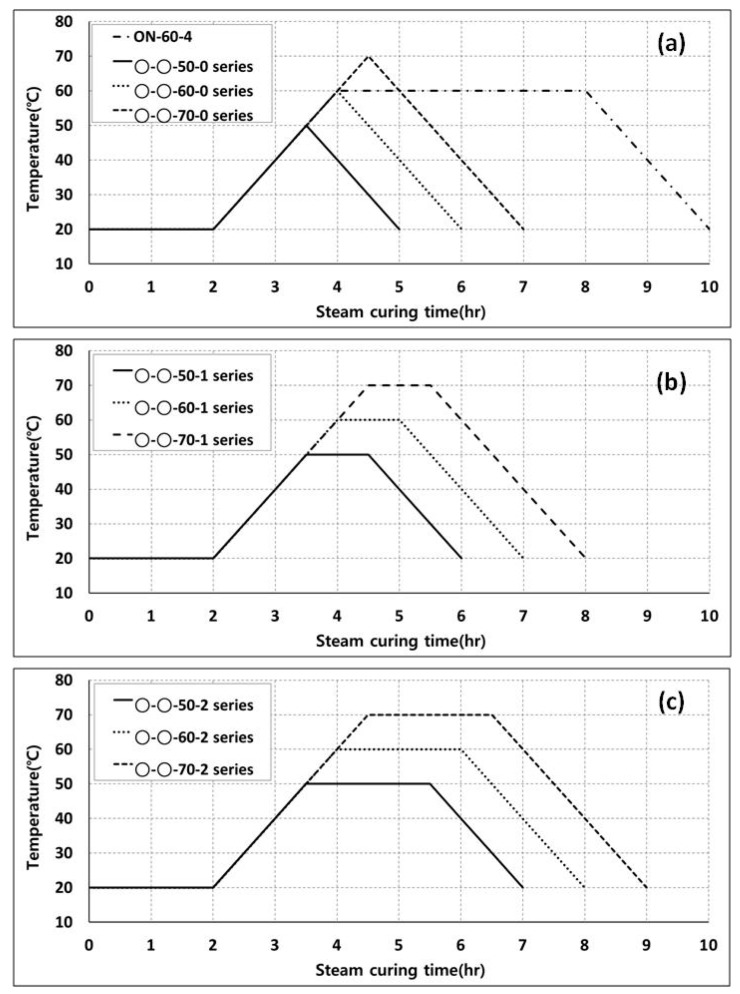
Steam-curing regimes employed in the experimental program; (**a**) control series and steam-curing regimen holding the maximum temperature for one hour, (**b**) steam-curing regimen holding the maximum temperature for one hour, and (**c**) steam-curing regimen holding the maximum temperature for two hours.

Specimens from all four aforementioned series were subjected to steam curing under varying conditions (Figure 3), e.g., the maximum steam-curing temperature and the maximum holding duration. The details of these parameters are set out in Table 3. Another mix (O-N-60-4) was also formulated for the conventionally used steam-curing regimen. The nomenclature stipulated in Table 3 refers to four parameters, namely cement type, aggregate type, the maximum temperature of the steam-curing cycle, and the duration of holding/maintaining the maximum temperature of the curing cycle. For instance, the mix ID M-R-70-2 refers to the concrete mix incorporating HESC as the primary binder, and the recycled aggregate blend (both for coarse and fine aggregates), with the maximum temperature of the steam-curing cycle set at 70 °C, maintained for two hours. The curing cycle was designed based on the empirical cycle length in accordance with the Portland Cement Association (PCA) [29]. Various parameters were employed in order to determine the optimum parameters based on the mechanical strength of the resulting concretes.

### 2.4. Experimental Methods and Procedures

**Figure 4:**
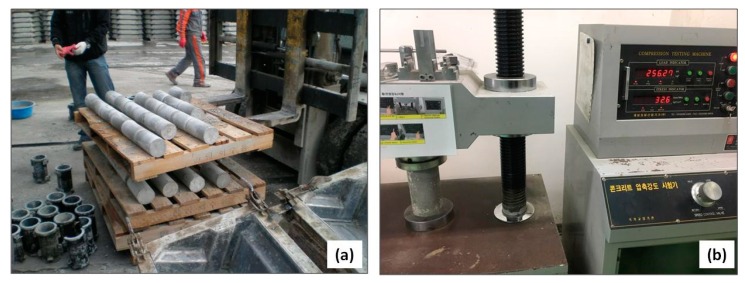
(**a**) Steam-cured samples being removed from molds, and (**b**) compression testing setup (testing was carried out on the cylindrical specimens according to ASTM C39 [42,45] and ASTM C469 [46]).

The properties of the developed concretes (both in fresh and hardened states) were evaluated by various ASTM testing standards [42,43,44,45,46]. Figure 4 shows various steam-cured samples being removed from the molds, and the compression testing setup. Fresh state properties were determined by a slump cone and digital air content meter (images are not shown due their common use in concrete testing). The specimens were tested at various curing ages, to assess their attributes at various hydration stages. The compressive strength testing was done by crushing the cylindrical specimen (100 mm diameter) in an automatic compression testing machine (5000 kN capacity), subjected to the loading rate of 2.4 kN/s [42].

## 3. Results, Discussion, and Analyses

### 3.1. Fresh State Concrete Properties

**Figure 5:**
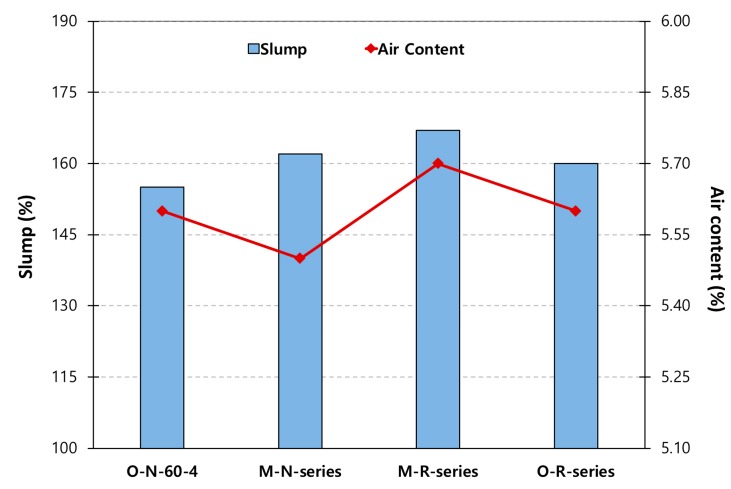
Fresh state properties (slump and air content).

The fresh state properties of the concretes are shown in Figure 5. Both the cement and aggregate types seemed to affect the results. Mixes containing HESC showed higher flowability, as indicated by the greater slump values. Similarly, the inclusion of a blend of recycled aggregates further increased the slump. The rheological properties (flowability/workability) of fresh RAC and NAC was similar to the previous findings [22,25,47] by the same authors. The rounded aggregates may upsurge/increase the workability of fresh concrete due to a “ball-bearing” effect [48]. As explained, such an effect can be attributed to the pretreatment (grinding, churning, and chemical processes) for adhered mortar removal, which may lead to the breaking of angular ends of aggregate particles, resulting in more spherical shapes [15].

The air content results were rather different to those obtained for the slump, as a general trend could not be ascertained. The air content tended to increase in the concretes that contained recycled aggregates. This can be explained by the poor packing of the somewhat rounded aggregate shape (of recycled aggregates), in contrast to the angular grains (of natural aggregates). The greater air content also signified an anticipated decline in compressive strength, due to a greater void ratio and lower gel/space ratio. As the present study primarily focused on the effect of varying steam-curing conditions, the positive (or negative) effects will be reflected in the hardened concrete (as can be seen from the compressive strength results). It should also be pointed out that these properties were also affected by the dosages of admixtures, if used in the mix [49]. It is, however, worth mentioning that the published literature on the fresh state properties of RAC contains disparities owing to the varying w/c ratio, mixing method, and most importantly, the bonded/adhered mortar with the recycled aggregate surface [50,51].

### 3.2. Compressive Strength

**Figure 6:**
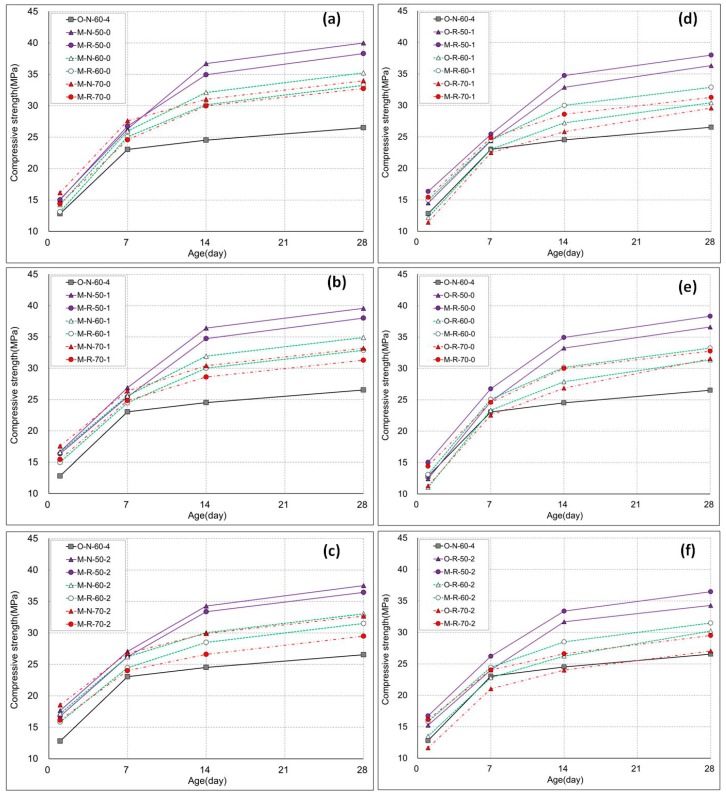
Compressive strength results of all of the mixes, in comparison with the control.

**Figure 7:**
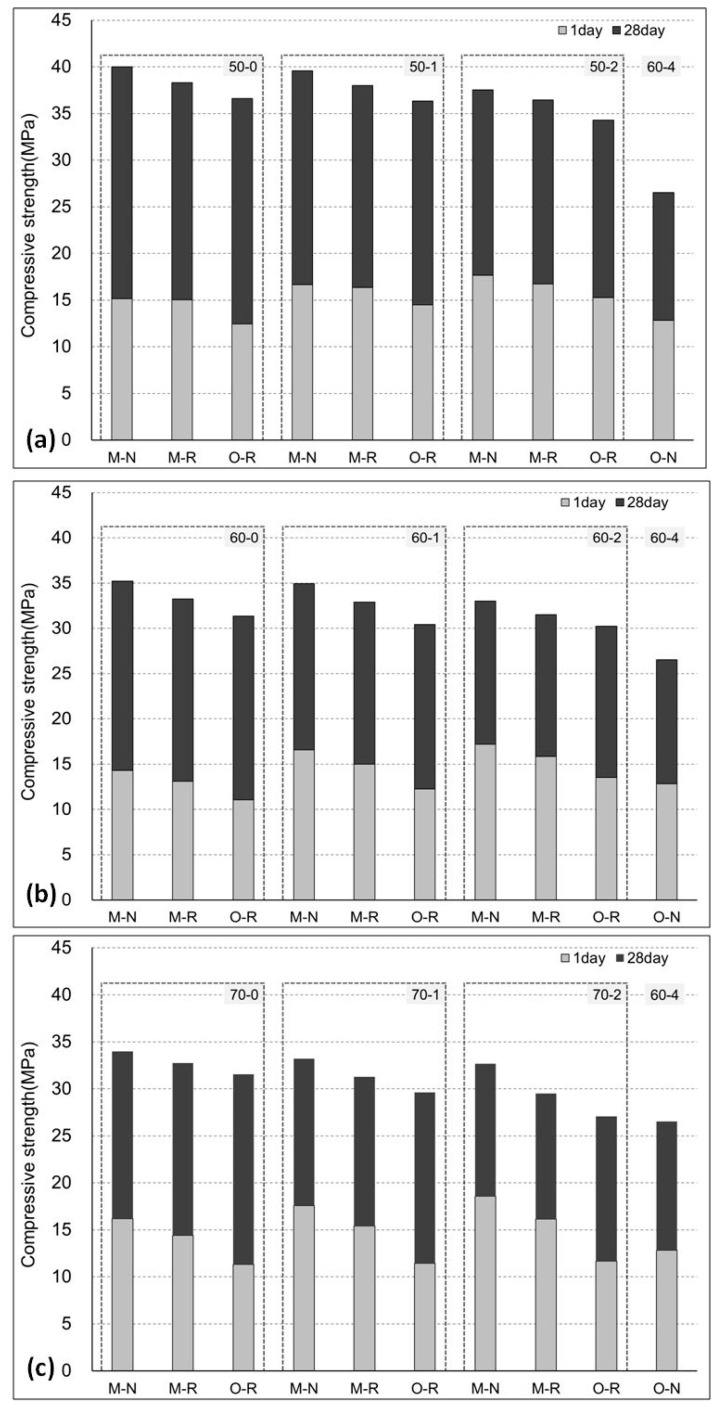
Comparison of the 1-day and 28-day strength of the concrete specimens with peak curing temperature of (**a**) 50 °C, (**b**) 60 °C, and (**c**) 70 °C.

The compressive strength results are shown in Figure 6 and Figure 7. The values are averages of three specimens, while the S.D/error was less than 4%. As steam curing was employed throughout, there was a need to determine the one-day strength of the hardened concrete specimens. Typically, strength increases with age due to the increased hydration, which can be easily seen in the results. Depending on the kind and amount of coarse and fine aggregate used, the HESC-incorporated concretes developed more than 50% of their design strength (30 MPa) at just one-day of age. This is a major strength improvement after just one day. On the other hand, the control reached almost 40% of the designed strength, owing to the lower hydration rate. The usefulness of using the moderately high early-strength cement is indicated by the disparity of strength attributes, in comparison to its counterpart. The reason for this enhanced strength gain stems from the fact that the HESC has a higher hydration rate, as corroborated by Hanif et al. [22]. This strength increase with age follows a logarithmic trend.

It was observed that the compressive strength of the specimens decreased as the maximum temperature of the steam-curing cycle increased. A great disparity in strength was observed when compared to the control mix (O-N-60-4). This is consistent with the maturity theory of concrete [52,53]. The maximum temperature for efficient curing under the same conditions, as used in this experiment, was estimated to be below 60 °C. The results are in agreement with the previous findings [54], where it was shown that the reduction in mechanical properties of the RAC was “more pronounced in water curing than in steam curing”. Also, the precast concrete elements with RA incorporation using steam curing have been proven to meet the strength and durability requirements [55].

Another steam-curing parameter evaluated in this study was the duration for which the maximum temperature was maintained. The experimental study demonstrated that a one-hour holding duration of the maximum temperature was optimum. Previously, Ramezanianpour et al. [26,27] had shown that for self-consolidating concrete and for concrete containing mineral admixtures, a lower duration of exposure to the maximum temperature was more beneficial. The experimental findings in the current study corroborate similar outcomes for RAC. This clearly shows that maintaining the peak temperature for a greater time period is not of increased benefit. Not only is the strength reduced, but more resources (energy and cost) are also expended, which hinders the sustainability goals. It is also imperative to specify the higher rate of shrinkage due to the very high temperature.

Typically, the increased temperature is thought to increase the early strength of concrete due to an escalated hydration reaction. However, this generalization cannot be guaranteed for a different cement type that has a moderately high early strength (as used in this study). Both the physical properties (fineness and surface area), and chemical composition, play a vital role in the rate of the hydration reaction. The fact that the tested concrete had a high compressive strength despite the recycled aggregate, low temperature steam curing, and short duration, may be due to the characteristics of the moderately high early strength cement. Conventionally-produced high early strength cement has a higher C3S content than ordinary Portland cement. Therefore, the early strength is high, and the hydration heat and drying shrinkage are increased, while the long-term strength is lower than that of ordinary Portland cement [37]. It is suggested that the lower content of C3S and the increased content of C2S in moderately high early strength cement lead to its improved attributes [24].

## 4. Conclusions

In this experimental study, the properties of steam-cured concrete were evaluated in an attempt to determine the optimum conditions of a steam-curing cycle for recycled aggregate concrete, incorporating high early strength cement (HESC). Concrete specimens containing ordinary Portland cement (OPC) and HESC were formulated. After specimen casting, varying conditions of steam curing were employed. The steam-curing cycle was set based on the peak temperature, and the duration for which the peak temperature was maintained. Three peak temperatures were used for steam curing, 50 °C, 60 °C, and 70 °C, maintained for up to two hours. The compressive strength results indicated that the steam-curing cycle employing the peak temperature of 50 °C and maintained for one hour with a total duration of six hours, was the optimum method for strength development, both at the early and late stages of hydration.

The following meaningful conclusions were drawn from this experimental study:

- Steam curing significantly increases the hydration rate of recycled aggregate concrete, thus helping to attain the target (design strength) within three days of casting.
- Utilizing moderately high early strength cement leads to a higher rate of strength gain of recycled aggregate concrete when steam curing is employed. This leads to increased early-age strength, without compromising the ultimate strength owing to the higher C2S content.
- The optimum conditions for steam curing based on the strength criterion were a heating/cooling rate of 20 °C/h and a peak temperature of 50 °C, maintained for one hour.
- Determining the optimum steam-curing temperature and duration will help reduce the associated curing costs, thus further economizing the production of recycled aggregate concrete.

The research findings point towards a useful and economical application for steam-cured recycled aggregate concrete in precast concrete construction, where the goal is speedy construction with superior mechanical properties. The use of recycled aggregates can slow the depletion of natural resources and help promote sustainable development. It is, however, clarified that the accelerated hydration for faster strength gain might lead to shrinkage and cracking, which must be evaluated prior to critical structural elements, particularly for structures with a higher ratio of surface area to depth, such as slabs and pavements. Nevertheless, the use of shrinkage-reducing admixtures or expansive cement can reduce the likelihood of such cracking.

Although the use of steam-cured RAC with optimum duration and temperature conditions ought to maximize the resulting mechanical attributes, the cost of achieving this and the associated carbon dioxide emissions must also be evaluated to determine the overall impact.

## Figures and Tables

**Table 1:** Physical characteristics of the aggregates used in the experiment.

Raw Materials		Maximum Particle Size (mm)	Specific Gravity	Water Absorption (%)
Coarse Aggregate	Natural	25	2.69	0.91
	Recycled		2.54	2.16
Fine Aggregate	Natural	5	2.60	0.92
	Recycled		2.44	5.17
Cement	Ordinary Portland	-	3.14	-
	Moderately High Early Strength		3.17	-

**Table 2:** Mixture proportions (all quantities in kg/m^3^ unless specified otherwise).

Variable	W/C	S/a	Unit Weight (kg/m^3^)					
			W	C	Fine Aggregate		Coarse Aggregate	
					Natural	Recycled	Natural	Recycled
O-N-60-4	36.4	43.8	155	425	751	0	998	0
M-N-series			155	429	751	0	998	0
M-R-series			155	429	451	282	399	565
O-R-series			155	425	451	282	399	565

**Table 3:** Mix details and steam-curing regimes employed.

Mix ID	Binder Type *	Coarse Aggregate		Fine Aggregate		Curing	
		Natural	Recycled	Natural	Recycled	M.T. **	T.D. ***
O-N-60-4	O	100%	0%	100%	0%	60 °C	4 h
M-N-50-0	M	100%	0%	100%	0%	50 °C	0 h
M-N-50-1							1 h
M-N-50-2							2 h
M-N-60-0						60 °C	0 h
M-N-60-1							1 h
M-N-60-2							2 h
M-N-70-0						70 °C	0 h
M-N-70-1							1 h
M-N-70-2							2 h
M-R-50-0		40%	60%	60%	40%	50 °C	0 h
M-R-50-1							1 h
M-R-50-2							2 h
M-R-60-0						60 °C	0 h
M-R-60-1							1 h
M-R-60-2							2 h
M-R-70-0						70 °C	0 h
M-R-70-1							1 h
M-R-70-2							2 h
O-R-50-0	O	40%	60%	60%	40%	50 °C	0 h
O-R-50-1							1 h
O-R-50-2							2 h
O-R-60-0						60 °C	0 h
O-R-60-1							1 h
O-R-60-2							2 h
O-R-70-0						70 °C	0 h
O-R-70-1							1 h
O-R-70-2							2 h

* Binder Type (O: Ordinary Portland Cement, M: Moderately High Early Strength Cement); ** M.T.: Maximum temperature during steam curing; *** T.D.: Time duration at maximum temperature.

## References

[1] De Schutter G. (2002). Fundamental study of early age concrete behaviour as a basis for durable concrete structures. Mater. Struct..

[2] Nixon P.J. (1978). Recycled concrete as an aggregate for concrete—A review. Matér. Constr..

[3] Xiao J. (2018). Recycled Aggregate Concrete.

[4] Bontempi E. (2017). A new approach for evaluating the sustainability of raw materials substitution based on embodied energy and the CO_2_ footprint. J. Clean. Prod..

[5] Lu Y., Chen X., Teng X., Zhang S. (2016). Dynamic compressive behavior of recycled aggregate concrete based on split Hopkinson pressure bar tests. Mater. Struct..

[6] De Brito J., Ferreira J., Pacheco J., Soares D., Guerreiro M. (2016). Structural, material, mechanical and durability properties and behaviour of recycled aggregates concrete. J. Build. Eng..

[7] Silva R.V., de Brito J., Dhir R.K. (2015). Establishing a relationship between modulus of elasticity and compressive strength of recycled aggregate concrete. J. Clean. Prod..

[8] Bogas J.A., De Brito J., Ramos D. (2015). Freeze–thaw resistance of concrete produced with fine recycled concrete aggregates. J. Clean. Prod..

[9] Silva R.V., Neves R., De Brito J., Dhir R.K. (2015). Carbonation behaviour of recycled aggregate concrete. Cem. Concr. Compos..

[10] Yehia S., Helal K., Abusharkh A., Zaher A., Istaitiyeh H. (2015). Strength and Durability Evaluation of Recycled Aggregate Concrete. Int. J. Concr. Struct. Mater..

[11] Matias D., De Brito J., Rosa A., Pedro D. (2014). Durability of Concrete with Recycled Coarse Aggregates: Influence of Superplasticizers. J. Mater. Civ. Eng..

[12] Lye C.Q., Dhir R.K., Ghataora G.S., Li H. (2016). Creep strain of recycled aggregate concrete. Constr. Build. Mater..

[13] Gonzalez-Corominas A., Etxeberria M. (2016). Effects of using recycled concrete aggregates on the shrinkage of high performance concrete. Constr. Build. Mater..

[14] Bendimerad A.Z., Rozière E., Loukili A. (2016). Plastic shrinkage and cracking risk of recycled aggregates concrete. Constr. Build. Mater..

[15] Kim Y., Hanif A., Kazmi S.M.S., Munir M.J., Park C. (2018). Properties enhancement of recycled aggregate concrete through pretreatment of coarse aggregates—Comparative assessment of assorted techniques. J. Clean. Prod..

[16] Zhang R., Cheng X., Hou P., Ye Z. (2015). Influences of nano-TiO_2_ on the properties of cement-based materials: Hydration and drying shrinkage. Constr. Build. Mater..

[17] Shah S.P., Hou P., Konsta-Gdoutos M.S. (2015). Nano-modification of cementitious material: Toward a stronger and durable concrete. J. Sustain. Cem. Mater..

[18] Sobolev K., Lin Z., Flores-Vivian I., Pradoto R. (2016). Nano-Engineered Cements with Enhanced Mechanical Performance. J. Am. Ceram. Soc..

[19] Jamshidi A., Kurumisawa K., Nawa T., Samali B., Igarashi T. (2017). Evaluation of energy requirement and greenhouse gas emission of concrete heavy-duty pavements incorporating high volume of industrial by-products. J. Clean. Prod..

[20] Türkel S., Alabas V. (2005). The effect of excessive steam curing on Portland composite cement concrete. Cem. Concr. Res..

[21] Park J.S., Kim Y.J., Cho J.R., Jeon S.J. (2015). Early-age strength of ultra-high performance concrete in various curing conditions. Materials.

[22] Hanif A., Kim Y., Lu Z., Park C. (2017). Early-age behavior of recycled aggregate concrete under steam curing regime. J. Clean. Prod..

[23] Liu B., Xie Y., Li J. (2005). Influence of steam curing on the compressive strength of concrete containing supplementary cementing materials. Cem. Concr. Res..

[24] Kim Y.-J., Kim S.-W., Park C.-W., Sim J.-S. (2016). Compressive Strength Properties of Concrete Using High Early Strength Cement and Recycled Aggregate with Steam Curing Conditions. J. Korean Recycl. Constr. Resour. Inst..

[25] Hanif A., Kim Y., Lee K., Park C., Sim J. (2017). Influence of cement and aggregate type on steam-cured concrete—An experimental study. Mag. Concr. Res..

[26] Ramezanianpour A.A., Khazali M.H., Vosoughi P. (2013). Effect of steam curing cycles on strength and durability of SCC: A case study in precast concrete. Constr. Build. Mater..

[27] Ramezanianpour A.M., Esmaeili K., Ghahari S.A., Ramezanianpour A.A. (2014). Influence of initial steam curing and different types of mineral additives on mechanical and durability properties of self-compacting concrete. Constr. Build. Mater..

[28] Ba M.F., Qian C.X., Guo X.J., Han X.Y. (2011). Effects of steam curing on strength and porous structure of concrete with low water/binder ratio. Constr. Build. Mater..

[29] Kosmatka S.H., Kerkhoff B., Panarase W.C. (2008). Design and Control of Concrete Mixtures.

[30] (2006). Standard Test Method for Sieve Analysis of Fine and Coarse Aggregate.

[31] (2006). Testing Method for Density and Absorption of Fine Aggregate.

[32] (2006). Testing Method for Density and Absorption of Coarse Aggregate.

[33] (2006). Recycled Aggregate for Concrete.

[34] (2002). Standard Practice for Selecting Proportions for Normal, Heavyweight, and Mass Concrete.

[35] (2008). Building Code Requirements for Structural Concrete.

[36] Lee H., Hanif A., Usman M., Sim J., Oh H. (2018). Performance evaluation of concrete incorporating glass powder and glass sludge wastes as supplementary cementing material. J. Clean. Prod..

[37] Kim Y., Hanif A., Usman M., Munir M.J., Kazmi S.M.S., Kim S. (2018). Slag waste incorporation in high early strength concrete as cement replacement: Environmental impact and influence on hydration & durability attributes. J. Clean. Prod..

[38] Hanif A. (2017). Recycled Aggregate Use in Precast Concrete: Properties & Applications.

[39] Hanif A., Kim Y., Lee H., Park C., Sim J. Suitability Assessment of Reinforced Precast Concrete Blocks Incorporating Recycled Aggregate. Proceedings of the Korea Concrete Institute Autumn Convention 2011.

[40] Hanif A., Kim Y., Kang T., Lee T., Jo C., Sim J. A Study on the Compressive Strength of Precast Concrete Using Recycled Aggregate Concrete. Proceedings of the Korea Concrete Institute Spring Convention 2011.

[41] Sim J., Park C., Park S., Kim Y. (2006). Characterization of Compressive Strength and Elastic Modulus of Recycled Aggregate Concrete with Respect to Replacement Ratios. J. Korean Soc. Civ. Eng..

[42] (2003). Standard Test Method for Compressive Strength of Cylindrical Concrete Specimens.

[43] (2003). Standard Test Method for Slump of Hydraulic-Cement Concrete.

[44] (2004). Standard Test Method for Air Content of Freshly Mixed Concrete by the Pressure Method.

[45] Li Z. (2011). Advanced Concrete Technology.

[46] (1994). Standard Test Method for Static Modulus of Elasticity and Poisson’ s Ratio of Concrete.

[47] Usman M., Khan A.Y., Farooq S.H., Hanif A., Tang S., Khushnood R.A., Rizwan S.A. (2018). Eco-friendly self-compacting cement pastes incorporating wood waste as cement replacement: A feasibility study. J. Clean. Prod..

[48] Neville A.M. (2004). Properties of Concrete.

[49] Rasheed A., Usman M., Farooq H., Hanif A. (2018). Effect of Super-plasticizer Dosages on Fresh State Properties and Early-Age Strength of Concrete. IOP Conf. Ser. Mater. Sci. Eng..

[50] de Brito J., Saikia N. (2013). Recycled Aggregate in Concrete Use of Industrial, Construction and Demolition Waste.

[51] Osborne G.J. (1999). Durability of Portland blast-furnace slag cement concrete. Cem. Concr. Compos..

[52] Wade S. (2005). Evaluation of the Maturity Method to Estimate Concrete Strength. Mater’s Thesis.

[53] Wedding P., Carino N. (1984). The Maturity Method: Theory and Application. Cem. Concr. Aggreg..

[54] Poon C.S., Chan D. (2006). Paving blocks made with recycled concrete aggregate and crushed clay brick. Constr. Build. Mater..

[55] Vázquez E. (2013). Progress of Recycling in the Built Environment.

